# Postorotracheal intubation dysphagia in patients with COVID-19: A retrospective study

**DOI:** 10.1590/1516-3180.2022.0608.R3.14032024

**Published:** 2024-05-24

**Authors:** Mariana Saconato, Jean Henri Maselli-Schoueri, Ceila Maria Sant’Ana Malaque, Rosa Maria Marcusso, Augusto César Penalva de Oliveira, Lucio Antonio Nascimento Batista, Graziela Ultramari, José Angelo Lauletta Lindoso, Maria Inês Rebelo Gonçalves, Jaques Sztajnbok

**Affiliations:** I PhD. Speech therapist, Technical manager of the Speech Therapy team, Instituto de Infectologia Emílio Ribas (IIER), São Paulo (SP), Brazil.; II MD. Physician , Centro Universitário Faculdade de Medicina do ABC (FMABC), Santo André (SP), Brazil.; III PhD. Physician, Intensive Care Unit Physician, Instituto de Infectologia Emílio Ribas (IIER), São Paulo (SP), Brazil.; IV MSc. Statistician, Instituto de Infectologia Emílio Ribas (IIER), São Paulo (SP), Brazil.; V PhD. Neurologist, Instituto de Infectologia Emílio Ribas (IIER), São Paulo (SP), Brazil.; VI Speech Therapist, Instituto de Infectologia Emílio Ribas (IIER), São Paulo (SP), Brazil.; VII MSc. Physiotherapist, Head of the Diagnostic and Therapeutic Support Department, Instituto de Infectologia Emílio Ribas (IIER), São Paulo (SP), Brazil.; VIII PhD. Physician, Director of the Diagnostic and Therapeutic Support Department, Instituto de Infectologia Emílio Ribas (IIER), São Paulo (SP), Brazil.; IX PhD. Speech therapist and Professor, Department of Speech Therapy, Universidade Federal de São Paulo (UNIFESP), São Paulo (SP), Brazil; X MD. Physician, Head of the Intensive Care Unit, Instituto de Infectologia Emílio Ribas (IIER), São Paulo (SP), Brazil.

**Keywords:** Deglutition, Intubation, COVID-19, Deglutition disorders, Rehabilitation, Post orotracheal intubation, Dysphagia, Swallowing, Tracheostomy

## Abstract

**BACKGROUND:**

The cause of oropharyngeal dysphagia in patients with coronavirus disease (COVID-19) can be multifactorial and may underly limitations in swallowing rehabilitation.

**OBJECTIVE:**

Analyze the factors related to dysphagia in patients with COVID-19 immediately after orotracheal extubation and the factors that influence swallowing rehabilitation.

**DESIGN AND SETTING:**

A retrospective study.

**METHODS:**

The presence of dysphagia was evaluated using the American Speech-Language Hearing Association National Outcome Measurement System (ASHA NOMS) scale and variables that influenced swallowing rehabilitation in 140 adult patients who required invasive mechanical ventilation for >48 h.

**RESULTS:**

In total, 46.43% of the patients scored 1 or 2 on the ASHA NOMS (severe dysphagia) and 39.29% scored 4 (single consistency delivered orally) or 5 (exclusive oral diet with adaptations). Both the length of mechanical ventilation and the presence of neurological disorders were associated with lower ASHA NOMS scores (odds ratio [OR]: 0.80, 95% confidence interval [CI]: 0.74–0.87 P < 0.05; and OR: 0.13, 95% CI: 0.61–0.29; P < 0.05, respectively). Age and the presence of tracheostomy were negatively associated with speech rehabilitation (OR: 0.92; 95% CI: 0.87–−0.96; OR: 0.24; 95% CI: 0.80–−0.75), and acute post-COVID-19 kidney injury requiring dialysis and lower scores on the ASHA NOMS were associated with longer time for speech therapy outcomes (β: 1.62, 95% CI, 0.70–3.17, P < 0.001; β: −1.24, 95% CI: −1.55–−0.92; P < 0.001).

**CONCLUSION:**

Prolonged orotracheal intubation and post-COVID-19 neurological alterations increase the probability of dysphagia immediately after extubation. Increased age and tracheostomy limited rehabilitation.

## INTRODUCTION

The most common and severe complication in patients with coronavirus disease 2019 (COVID-19) is the acute respiratory distress syndrome (ARDS), with acute respiratory failure as the main cause of hospitalization and orotracheal intubation (OTI) in intensive care units (ICU).^1-4^ Consequently, several studies have shown an OTI rate ranging from 12% to 33% of hospitalized patients while the time they remained intubated also varied but deserves attention with >50% of those patients needed mechanical ventilation for up to 14 days.^5-8^ Thus, the longer the time under OTI, the greater the chances of mechanical and sensitive sequelae.^9-16^

Therefore, the longer the length of mechanical ventilation, the greater the chances that the patient will present lesions in the oropharyngolarynx region, vocal fold paresis or paralysis, supraglottic edema, arytenoid dislocation, granulomas, and infraglottic strictures in addition to weakness in the base of the tongue and pharynx muscles and desensitization of sensory receptors in the tongue, pharynx, and larynx,^12-19^ all of which could contribute to or even cause dysphagia.

## OBJECTIVE

Thus, considering the high prevalence of dysphagia, the present study analyzed the risk factors associated with dysphagia onset in patients with severe COVID-19 who were administered OTI for >48 h in the ICU.

## METHODS

### Study design and inclusion/exclusion criteria

This was a retrospective cohort study conducted in the ICU of a reference center for the care of infectious diseases, which has exclusively received suspected and confirmed cases of COVID-19 since March 2020.

Initially, 161 patients who underwent invasive mechanical ventilation for >48 h were considered for this study. However, 15 patients with previous neurological diseases and six patients with a diagnosis of and treatment for head and neck cancer were excluded because these patients could already present pre-existing alterations in swallowing biomechanics. In total, 140 patients were included in the final sample.

All patients included in this study required OTI for >48 h and were evaluated by speech therapists according to the institutional protocol.

Therefore, 140 adult patients who were admitted to the ICU between March and June 2020 and who had positive reverse transcription PCR (RT-PCR) results for severe acute respiratory syndrome coronavirus 2 (SARS-CoV-2) were considered eligible for the study.

### Variables

In addition to the time of OTI, complications developed by the patients were observed and categorized as: neurological, defined as persistent delirium, ischemic and/or hemorrhagic strokes, encephalitis, and encephalopathies; cardiac, confirmed by clinical criteria, imaging, and laboratory tests, such as increased levels of troponin, myoglobin, C-reactive protein, serum ferritin, and interleukin-6;^20,21^ or renal, defined as the need for hemodialysis in individuals without a history of chronic kidney disease. All alterations were diagnosed and confirmed by critical care physicians. The prone position, a ventilatory support strategy to increase oxygenation levels by reducing the ventilation/perfusion ratio, was considered in this study only when invasive mechanical ventilation was required.

### Speech therapy assessment

Speech therapy assessment of swallowing is routinely performed in the ICU of the Instituto de Infectologia Emílio Ribas (IIER). Before the pandemic, all patients undergoing prolonged OTI were evaluated 24 h after extubation. However, this changed for patients with SARS-CoV-2 and for 48 h afterwards. Thus, all 140 patients included in this study were evaluated by a speech therapy team 48 h after extubation.

This change was justified by the severity of the pulmonary condition associated with possible extubation failures, which can occur within 72 h after orotracheal extubation and is more common in patients diagnosed with pulmonary diseases.^14^ Another factor that determined the postponement of the evaluation was the presence of residual sedation that could interfere with the evaluation findings^12^ since many patients with COVID-19 require high doses of analgesia and neuromuscular blockers to maintain respiratory synchrony during mechanical ventilation.

After analyzing the electronic medical records for a survey of demographic, clinical, and laboratory information, a speech-language evaluation of swallowing was initiated.

### Swallowing assessment procedures

The oral sensory-motor system was evaluated, which included the assessment of the strength and mobility of the lips and tongue, contraction of the masseter muscles during mastication, soft palate and mandible mobility, hyolaryngeal complex elevation and support, and vocal quality before food offering.

The functional evaluation of swallowing consisted initially of the offer of pasty food, followed by a thickened liquid with honey and nectar consistencies, liquids without thickeners, and finally, semi-solid and dry solids.

Food volumes ranged from 3 to 100 mL for the pasty food, offered by a spoon; 3 to 180 mL for thin and thickened liquids offered in 3 and 5 mL scoops, controlled and free sips; a portion of soft solid (small roll) and dry solid (cream cracker).

The volume and consistency provided during the evaluation progressed according to the findings of the oral and pharyngeal phases of swallowing. Important changes in the preparatory and oral phases of swallowing homogeneous pasty and thickened liquids make it impossible to offer liquids without thickeners or semi-solids and solid foods.

The significant drop in peripheral oxygen saturation due to the removal of the nonrebreather mask also caused the interruption of the functional assessment of swallowing, as well as the need to remove the speech valve and reinflate the cuff in tracheostomized patients who had respiratory distress.

The American Speech-Language Hearing Association National Outcome Measurement System (ASHA NOMS)^22^ was used to determine the level of swallowing function after bedside assessments. the patients were divided into three groups according to the ASHA scale score for tube feeding, moderate dysphagia, and minimal dysphagia (ASHA-NOMS scores of 1–3, 4–5, and 6, respectively).

The IIER ICU beds have a negative-pressure system. Despite this, all speech therapists performed speech therapy evaluations using personal protective equipment: private clothing, disposable aprons, caps, goggles, N95 masks, face shields, and gloves. All recommendations for dressing and undressing steps provided by the Hospital Infection Control Commission were followed.

### Swallowing assessment procedures in tracheostomized patients

Due to the inaccessibility of RT-PCR tests to confirm the negative status of the patients, the speech-language evaluation was performed even in tracheostomized patients. For this population, it was initially stipulated that the assessment with cuff deflation and adaptation of the Passy–Muir phonatory valve or occlusion of the tracheostomy should be performed at least 25 days after the positive RT-PCR result. Until May 2020, no scientific evidence was available for the virus transmission time.

From May 2020 onwards, the evaluation protocol for tracheostomized patients was modified once again as one study showed that a sharp drop occurred in infecting viral particles and antibody growth after 20 days. Thus, tracheostomized patients were evaluated 20 days after positive RT-PCR results were obtained.^2^

### Statistical analysis and ethical aspects

The prespecified outcome variables were the time to discharge from speech therapy, time spent under OTI, ASHA scale score 48 h after extubation, successful rehabilitation (yes or no), and oral diet onset time.

For cases where time was considered a dependent variable, multiple linear regression was used; thus, three models were created: 1) time to discharge from speech therapy, 2) oral diet onset time, and 3) time spent intubated. Logistic regression was used for both situations in which the outcome variable was either binary (simple logistic regression for successful rehabilitation) or ordinal (ordinal logistic regression for the ASHA NOMS scale after 48 h of extubation). A stepwise backward strategy with the withdrawal of variables at 0.05 was used for all five models.

The confidence interval (CI) was 95% and the statistical program used was Stata (Stata Corp., College Station, United States) 12.0. This study was approved by the IIER Ethics Committee (protocol number 4.168.189).

## RESULTS

**Figure 1:**
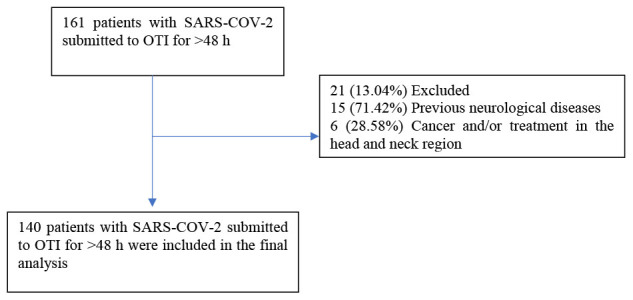
Sample selection according to inclusion and exclusion criteria.

Initially, 161 patients were eligible for inclusion. However, 21 patients were excluded from the sample because they presented with alterations in swallowing before the OTI (Figure 1).

SARS-CoV-2 = severe acute respiratory syndrome coronavirus 2.

Table 1 shows that the average age of patients evaluated after orotracheal extubation was 55.58 years with a predominance of females (50.71%). Systemic arterial hypertension was the most frequent comorbidity (46.43%), followed by obesity (42.86%) and diabetes mellitus (35%). As IIER is a reference in the care of infectious or contagious diseases, 2.86% of patients presented with the human immunodeficiency virus.

**Table 1:** Population, clinical characteristics and American Speech-Language Hearing Association National Outcome Measurement System scale and its outcomes after assessment and speech therapy discharge

Demographic and clinical variables	Total (n = 140)	(%)
Age		
Minimum–maximum	18–85	-
Median	57.5	-
Average ± standard deviation	55.58 ± 14.32	-
**Gender**		
Female	71	50.71
Male	69	49.29
**Previous comorbidities**		
Systemic arterial hypertension	65	46.43
Obesity	60	42.86
Diabetes mellitus	49	35.00
Heart disease	24	17.14
Chronic kidney injury	13	9.29
Human immunodeficiency virus	4	2.86
Smoking	28	20.00
**Orotracheal intubation and complications**		
**Length of mechanical ventilation**		
Minimum–maximum	2.0–33.0	-
Median	11	-
Average ± standard deviation	12.05 ± 6.07	-
**Reintubation**	30	21.43
**Tracheostomy**	23	16.43
**Prone position**	68	48.57
**Acute complications of COVID-19**		
Acute kidney injury requiring dialysis	37	26.43
Cardiomyopathies	17	12.14
Neurological disorders	58	41.43
**ASHA NOMS scale after speech therapy assessment**		
**Tube feeding**		
ASHA 1, ASHA 2	65	46.43
**Moderate dysphagia**		
ASHA 4, ASHA 5	55	39.29
**Minimal dysphagia**		
ASHA 6	20	14.29
**Speech-language therapy outcome**		
Rehabilitated	93	66.43
Not rehabilitated	47	33.57
**Time of onset for oral diet**		
Minimum–maximum	1.0–15.0	-
Median	3.0	-
Average ± SD	4.4 ± 2.81	-
**Average time for speech therapy discharge (days)**		
**Tube feeding (ASHA 1, ASHA 2, and ASHA 3)**		
Minimum–maximum	2.0–26.0	-
Median	7.0	-
Average ± SD	8.55 ± 5.26	-
**Moderate dysphagia (ASHA 4 and ASHA 5)**		
Minimum–maximum	1.0–3.0	-
Median	1.0	-
Average ± SD	1.03 ± 0.27	-

COVID-19 = coronavirus disease 2019, SD = standard deviation; ASHA NOMS = American Speech-Language Hearing Association National Outcome Measurement System

The patients remained under OTI for an average time of 12.05 days with a standard deviation ranging from 2 to 33 days. Of the 140 patients, 30 patients (21.43%) required reintubation within 72 h after extubation while tracheostomy was performed in 23 patients (16.43%). In addition, a prone position maneuver was required for 68 patients (48.57%) while they were still intubated. During the ICU stay, 27 patients (26.43%) developed acute kidney injury and required hemodialysis while 58 (41.43%) had neurological disorders. Cardiac abnormalities after COVID-19 were observed in 17 (12.14%) patients.

The number of patients with severe or moderate dysphagia reached 46.43%, and the nasogastric tube was maintained as the exclusive feeding route for these patients; 39.29% of patients needed significant adaptations, such as the ingestion of homogeneous pasty foods and thickened hydration.

Speech therapy evaluation was performed 48 h after extubation, and 65 patients (46.43%) presented with severe dysphagia and needed to maintain the nasoenteral tube as an exclusive alternative to feeding, with ASHA scores of 1 and 2. In addition to clinical signs of penetration/aspiration, such as coughing, gagging, and changes in vocal quality shortly after swallowing, these patients showed significant fatigue when the nonbreathing mask was removed, further increasing the risk of bronchoaspiration during and after swallowing owing to a lack of coordination between swallowing and breathing.

No patient was rated on the ASHA NOMS level 3 scale; 55 patients (39.29%) had an exclusive oral diet with restrictions on consistency and the use of compensatory strategies, and 20 (14.29%) had minimal changes or normal swallowing, without the need for modifications and/or compensatory strategies during feeding.

Among the patients with dysphagia in whom the ASHA NOMS scale ranged from 1 to 5, 93 (66.43%) were rehabilitated while 47 (33.57%) were not. The average time for reintroduction of the oral diet in patients who needed to remain on an exclusive nasoenteral tube after the evaluation was 3 days.

Speech therapy discharge, with complete swallowing recovery, was achieved with an average of 8.55 days in patients with an initial ASHA NOMS score of 1–3, and 1.03 days in patients with an initial ASHA NOMS score of 4–5.

In the ordinal logistic regression, considering the values of the ASHA NOMS scale as the dependent variable, we found that both intubation time and the presence of neurological disorders were associated with lower scores on the ASHA NOMS scale (odds ratio, [OR]: 0.80, 95% CI: 0.74–0.87 P < 0.05; and OR: 0.13, 95% CI: 0.61–0.29 P < 0.05, respectively). Conversely, an inverse association was present for those patients who had been pronated (OR: 3.24; 95% CI: 1.51–6.94, P < 0.005) (Table 2).

**Table 2:** Multivariate models for variable analysis: ASHA NOMS scale, rehabilitation and average time for speech therapy discharge

Model 1: Ordinal logistic regression		ASHA NOMS scale				P value
		Z		OR	95% CI	
**Length of mechanical ventilation**		-5.36		0.80	0.74–0.87	< 0.001
**Prone position**		3.04		3.24	1.51–6.94	0.002
**Acute complications of COVID-19**						
Neurological disorders		-4.99		0.13	0.06–0.29	< 0.001
**Model 2: Logistic regression**		**Rehabilitation**				**P value**
		**Z**		**OR**	**95% CI**	
Age		-3.48		0.92	0.87 - 0.96	0.001
Tracheostomy		-2.45		0.24	0.80 - 0.75	0.014
**Model 3: Multivariate linear regression**	**Average time for speech therapy discharge (days)**					**P value**
	**β**		**R²**	**95% CI**		
Acute complications of COVID-19						
Acute kidney injury requiring dialysis	1.62		0.38	0.70–3.17		0.001
**ASHANOMS scale**	-1.24		0.38	(-1.55)–(-0.92)		0.001

ASHA NOMS = American Speech–Language–Hearing Association’s National Outcome Measurement System; OR = odds ratio; CI = confidence interval; COVID-19 = coronavirus disease 2019.

We found that both age and the presence of tracheostomy were negatively associated with speech rehabilitation (OR, 0.92: 95% CI: 0.87–0.96; OR, 0.24, 95% CI: 0.80–0.75), respectively. Finally, higher ASHA NOMS scores were negatively associated with the time needed for speech therapy discharge (β: −1.24; 95% CI: −1.55–−0.92; P < 0.001) (Table 2).

## DISCUSSION

Dysphagia after orotracheal intubation is multifactorial but is strongly related to the length of invasive mechanical ventilation. The presence of dysphagia can increase the time to reintroduction of an oral diet and the total hospital stay.

Herein, 46.43% of patients had severe dysphagia when assessed 48 h after extubation by the speech therapy team. The mean time to oral diet reintroduction was 4.4 days.

The prolonged duration of orotracheal intubation, which was an average of 11 days in this study, and the presence of acute complications of COVID-19 were related to severe dysphagia in the study population. Additionally, older patients and those who required tracheostomy had the greatest therapeutic limitations.

Some patients with severe COVID-19 can rapidly progress to ARDS, necessitating OTI and intensive care. In this sense, studies have shown that these patients could remain intubated between 7 and 14 days,^23^ which could be extended to >20 days in unfavorable cases.^24-26^ Thus, our study showed that for the 140 patients who were intubated, the average time spent under OTI was 12.05 days and the reintubation rate was 21.43%, which is slightly higher than that reported in specialized literature.^5^

One of the risk factors for dysphagia is the time spent under OTI, which is considered long if it exceeds 48 h.^22-24^ However, in addition to prolonged OTI, other conditions may justify the presence of changes in the swallowing biodynamics in ICU patients; these conditions include previous comorbidities and poor scores on disease severity scales, namely the Acute Physiology and Chronic Health Evaluation IV and the Simplified Acute Physiology Score II.^27-29^ Similarly, we found that many of our patients not only presented long periods under OTI but also had comorbidities before ICU admission.

No consensus is present in the literature regarding the ideal time to evaluate patients after orotracheal extubation. Although the average time for evaluation was 24 h;^27-31^ for the present study, all patients were evaluated 48 h after extubation.

Even after 48 h of extubation, 46.43% of patients in the present study had severe dysphagia, with lower scores on the ASHA NOMS scale (OR: 0.80; 95% CI: 0.74–0.87 P < 0.05) and prolonged orotracheal intubation time was directly related to these findings (Table 2).

Studies that evaluated swallowing after extubation in patients with COVID-19 showed dysphagia rates of 20%–90%, and dysphagia was attributed to prolonged mechanical ventilation time, age >60 years, duration of analgesia and neuromuscular blocker use, and the presence of tracheostomy.^32-34^

Information regarding the incidence of dysphagia in extubated patients without COVID-19 varies. This is mainly because of the different diagnostic methods and inconsistent evaluation intervals after extubation. In patients intubated for >48 h, the prevalence of dysphagia increases by 56%.^35^ According to other studies, dysphagia occurs in 3%–62% of patients recovering after critical illness^35^ and one-third of patients intubated after ARDS have dysphagia upon discharge from the hospital.^36^

Prolonged intubation contributes to the reduction of strength and mobility in both the lips and tongue and may contribute to the reduction of sensitivity of the tongue, pharynx, and larynx. Patients may present with increased oral transit time, delay in starting pharyngeal swallowing, reduced elevation of the hyolaryngeal complex during swallowing, and stasis in the oral cavity and hypopharynx after swallowing.^11,12,37,38^ In addition to motor and sensory failures, these patients may present with supraglottic edema, dislocation of the arytenoid cartilages, and paralysis or paresis of the vocal folds.^39,40^ Nevertheless, most of our patients still presented ASHA NOMS scores <6, thus requiring dietary alternatives, whether through feeding restrictions or supplementary feeding routes.

In addition to the extended period under OTI and the rate of reintubation, almost half of the patients included in this study were pronated. This is a ventilatory support strategy for increasing oxygenation levels through a reduction in the ventilation/perfusion ratio that has become more frequent during the COVID-19 pandemic.^41^

We previously hypothesized that prone positioning would decrease ASHA NOMS scores because of possible laryngeal lesions. However, we later found that the prone position was positively associated with ASHA NOMS scores. We also found no meaningful association between OTI time and maneuver, despite their tendency to be positively associated. Thus, these findings may be justified by the possible absence of laryngeal lesions in these patients given that several of these patients benefited from prone positioning, which may have reduced the total time spent on OTI.

Tracheostomy was performed in 16.43% of patients who were difficult to wean from mechanical ventilation. Following the speech therapy protocol, the speech therapist was recommended to assess these patients after at least 25 days elapsed since a positive RT-PCR result. However, as many patients remained on pressure-controlled mechanical ventilation for a long time, most started to meet the criteria for speech therapy 30 days after a positive RT-PCR result.

Tracheostomized patients did not tolerate the Passy–Muir speech valve for a prolonged period and presented with respiratory discomfort and decreased peripheral oxygen saturation. Severe dysphagia was present mainly in tracheostomized patients who developed ischemic and hemorrhagic stroke, both of which were defined as neurological complications herein. The strength, mobility, and sensitivity of the oral cavity and oropharyngeal structures was reduced, which contributed to the classification of these patients as having a score of 1 on the ASHA NOMS scale.

The difficulty in maintaining the Passy–Muir phonatory valve and the cannula occlusion for prolonged periods also contributed negatively to the rehabilitation of these patients as shown in the multivariate analysis in Table 2 (OR: 0.24; 95% CI: 0.80–0.75). This was because the reduction of subglottic pressure in tracheostomized patients can affect the time of lower airway closure and reduce the efficiency of cough.

In our sample, many patients had hypoactive delirium, whereas others had other neurological and cardiac disorders as well as renal disorders requiring hemodialysis. This combination of factors could have played a role in the number of nonrehabilitated patients (33.7%) and the average time (8.55 days) found for speech therapy discharge in patients with scores of 1 or 2 on the ASHA NOMS scale.

Regarding COVID-19 sequelae and other disorders, the virus exhibits neurotropic behavior with the capacity to invade the central nervous system. In addition, studies have shown that patients with acute kidney damage secondary to COVID-19 can present with important changes in fluid balance, with hemodynamic changes being common in this population. In our study, the presence of neurological disorders (stroke or delirium) was negatively associated with the ASHA NOMS scores. In addition, patients with acute kidney injury requiring dialysis spent more time on OTI.

Age is another factor contributing to the rehabilitation of patients with dysphagia. The results in Table 2 show that increasing age was negatively associated with rehabilitation success. This finding is also supported by current literature, which shows that disorders in swallowing biodynamics due to increasing age may be related to sarcopenia, sensory changes, and muscle weakness.^13,18^

Finally, although 46.43% of the patients had a score of 1 or 2 on the ASHA NOMS scale after the speech-language evaluation, the average time of onset of oral diet was 4.4 days. However, this does not mean that these patients were completely rehabilitated during this period as the average time to speech therapy discharge was 8.55 days.

Recent research has shown that patients with COVID-19 who require mechanical ventilation have the heaviest impact on quality within one year after discharge, with an increase in cardiovascular events, dyspnea, and rehospitalizations.^42^ Sarcopenic dysphagia resulting from the considerable loss of muscle mass during the ICU stay may be present in this group, as well as respiratory and vocal complaints related to possible late tracheal stenosis secondary to prolonged orotracheal intubation.

The present study had several limitations. Prolonged orotracheal intubation, reintubation, tracheostomy, and neurological, cardiac, and renal complications in individuals who develop severe forms of COVID-19 are factors that reinforce the multifactorial causes of dysphagia in these patients. However, other important variables were not analyzed in this study but also deserve attention, such as the period in which the patient remains sedated and pronated and the evaluation of sedoanalgesia (both drug and dose needed to achieve the desired effect), which might cause possible glottic lesions compromising speech and swallowing safety and causing persistent delirium, respectively. Notably, that the severity of COVID-19 at hospital or ICU admission was not considered in this study because of a lack of data.

## CONCLUSION

Prolonged orotracheal intubation and neurological alterations acquired after COVID-19 infection increase the probability of dysphagia immediately after extubation. Increasing age and the need for tracheostomies have limited the rehabilitation of these patients.
